# Reconstructing Organophosphorus Pesticide Doses Using the Reversed Dosimetry Approach in a Simple Physiologically-Based Pharmacokinetic Model

**DOI:** 10.1155/2012/131854

**Published:** 2012-02-01

**Authors:** Chensheng Lu, Leo Andres

**Affiliations:** ^1^Department of Environmental Health, Harvard School of Public Health, 401 Park Drive, Boston, MA 02215, USA; ^2^Atlanta Clinical and Translational Science Institute, School of Medicine, Emory University, 1440 Clifton Road, Atlanta, GA 30322, USA

## Abstract

We illustrated the development of a simple pharmacokinetic (SPK) model aiming to estimate the absorbed chlorpyrifos doses using urinary biomarker data, 3,5,6-trichlorpyridinol as the model input. The effectiveness of the SPK model in the pesticide risk assessment was evaluated by comparing dose estimates using different urinary composite data. The dose estimates resulting from the first morning voids appeared to be lower than but not significantly different to those using before bedtime, lunch or dinner voids. We found similar trend for dose estimates using three different urinary composite data. However, the dose estimates using the SPK model for individual children were significantly higher than those from the conventional physiologically based pharmacokinetic (PBPK) modeling using aggregate environmental measurements of chlorpyrifos as the model inputs. The use of urinary data in the SPK model intuitively provided a plausible alternative to the conventional PBPK model in reconstructing the absorbed chlorpyrifos dose.

## 1. Introduction

A physiologically based pharmacokinetic (PBPK) model would allow for simulating the dynamics of pesticide absorption, distribution, metabolism, and elimination (ADME) from different routes of exposures and, in theory, could be used as a tool for evaluating biomarker measurements (e.g. blood or urine levels) associated with the exposures [1–4]. The mechanistic representation of biological processes embedded in the PBPK model allows systematic route, dose, and species extrapolation, and for these reasons, PBPK models have been applied in pesticide risk assessments that are relevant for the interpretation of biomarker data [5–12].

Although the interpretation of PBPK model outputs could provide a link to the regulatory metrics in the form of reference dose, its application remains problematic due to the fundamental limitations resulting from the potential measurement errors associated with the aggregate exposure measurements [11, 13]. Although those aggregate exposure data are needed in order to simulate the dynamics of ADME for a specific pesticide, the uncertainties, mainly the temporal and spatial variations, associated with the measurements may inadvertently be carried over to the model outcomes. Those uncertainties, however, are merely explicit statements of underlying assumption applied in the analysis of urinary biomarker data.

A simple pharmacokinetic (SPK) model incorporating reverse dosimetry and PBPK modeling approach, on the other hand, only requires urinary biomarker data as inputs and the a-priori knowledge of the exposure pathways for individuals [14, 15]. The use of urinary biomarker data in the SPK simulation might be advantageous over the traditional PBPK model because urinary excretion is a primary route of elimination for many compounds, and urine samples are relatively easy to collect from individuals comparing to the aggregate environmental samples. The SPK model allows for the estimation of absorbed dose from a dominated route of exposure while reducing the number of inputs into the model and could potentially minimize the uncertainties. Therefore, the development of SPK model may be important in the field of PBPK model by reducing the resources needed to model the dose metrics of certain chemicals.

In this paper, we illustrated the development of an SPK model adapted from a previously published PBPK model and the performance of the exploratory analysis using urinary biomarker data as inputs to the SPK model. The effectiveness of the SPK model in the pesticide risk assessment was evaluated by comparing the dose estimates among three different composites of biomarker data. 

## 2. Methods

The development of the SPK model began with the pharmacokinetic equations that are used in previously developed PBPK models for animal and human data [2, 11, 16, 17]. While the solution of a conventional PBPK model results in a uniquely determined excretion profile of urinary biomarker, the “reverse” dosimetry analysis in this SPK model development, which uses urinary biomarker concentrations as input, does not lead to a unique solution. It is, therefore, necessary to constrain the dose profile with additional information to reach an optimum dosing profile. We assumed that the predominant exposure pathway for organophosphorus (OP) pesticides is via foods and, therefore, constructed the SPK model focusing on oral ingestion. Such assumption was based on the results of aggregate exposure assessment, and the previous PBPK model outputs which shows very little OP residues in the environment [18] and suggests dietary intakes of OP pesticides constitute the majority of urinary metabolite concentrations [11], respectively. 

### 2.1. Urinary Biomarker Data

**Figure 1:**
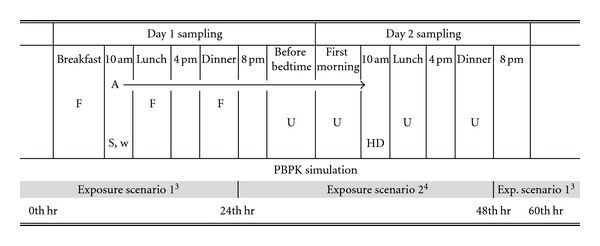
Sampling schedule for the 1998 study^1^ and the corresponding PBPK model simulation^2^.

Data employed in this SPK model simulation were collected from a cross-sectional study with repeated biological and environmental sampling conducted in Washington State in 1998. Results for the environmental measurements of several OP pesticides and their respective urinary metabolites, as well as for absorbed dose reconstruction using a conventional PBPK model, were published previously [11, 18–20]. In brief, the study was conducted in the homes of 13 children ages 3–6, who either lived in an urban/suburban (nonagricultural) area or in the agricultural region in which OPs have been used in the nearby fruit tree orchards. Each home was sampled for two 24-hour periods (over 3 days) in summer and fall of 1998. Environmental and biological sample collections included 24-hour indoor air, drinking water; outdoors soil, house dust, toy wipes, 24-hour duplicate diets, and 4 spot urine samples over a 24-hour period (Figure 1). The 1998 study was designed to capture the aggregate OP exposures in the subsequent 4 spot urines over the course of a 24-hour period. These were the before bedtime voids on the first day and the first morning lunch, and dinner voids on the second day. Chlorpyrifos (CPF) was selected as the modeled pesticide in this study because it was commonly detected in the environmental matrices, and its specific metabolite, 3,5,6-trichlorpyridinol (TCPY), was frequently measured in the urine samples, as compared to other OP pesticides. 

Urinary data from the 1998 study, including TCPY concentrations, urine void volumes, and urine sample collection times, were used as inputs into the SPK model to estimate the absorbed dose of CPF. The study protocol and procedures to obtain the assent of the children and informed consent of their parents or guardians were reviewed and approved by the University of Washington Institutional Review Board (IRB). The use of those data in the PBPK model analysis was reviewed and approved by Emory University IRB.

### 2.2. Model Development and Validation

#### 2.2.1. Input File Construction

A database was created in Microsoft Access 2002 to hold study variables and data. Tables in the database were structured in the same form as the raw study data to allow for easier manipulation of the data later in the analysis. There were missing values (not collected), including the void and exposure times, values for void volumes, and values for the weight of the study subjects that were necessary to create the data file. Values for the default void and exposure times were chosen based on a previous data file for the Matlab PK model script. Values for the default void volume were taken from a study by Voorhess [21] which examined urinary catecholamine excretion by healthy children. Lastly, values for the default weights of children were taken from table 7-3 of the Exposure Factors Handbook [22]. Tables were created to hold these values specific to sample, age, or sex depending on the measure. 

Queries were written in SQL to create the data files for input into the SPK model. SQL was used to join data from separate data tables into one query output, to make logic and arithmetic transformations to the data for input into the model, and to allow for dynamic creation of data files simplifying modification of data input file creation. Since some of the data that are necessary to create the input file were missing, default values had to be calculated. This was done according to the type of data that was available and which data would provide values that would best represent the inputs. The script would first try to determine values based on data that would yield the most representative values. If this data was not available, then it would try other data that would yield less representative values successively based on availability of the relevant data until it reached the least representative values.

The main query that produced the data file was qry_createdata. This query pulled data from database tables and other queries. These queries were qry_calcttime and qry_urinesampcalc. The qry_calcttime query was used to calculate the previous and current void times using available data and default values depending on the data that was available. The qry_urinesampcalc query was used to calculate the urinary excretion rate from metabolite concentrations, sample void volumes, and times from qry_calcttime. Once these queries produced the correct data for input into the model, the main query was used to export the data from Access into Excel 97–2002 format.

Once constructed, each of the input file for the Matlab SPK model script (see supplementary material available online at doi:10.1155/2012/131854) had nine variables. The first variable held the subject ID and study period. The subject ID was the part before the separator, and the study period was the part after the separator. For the study period, 1 signified the summer period, and 2 signified the fall period. For example, the identifier 114.1 denoted subject ID 114 in the summer study period. The second variable reflected whether the subject resided in an agricultural area. The third variable held the average metabolite urinary excretion rate that was calculated using the urinary metabolite concentration, the volume of the void, and the times for the previous and current void

(1) $${\text{UER}}_{\text{avg}}\;\text{(mmoL/hr)}\;=\frac{C_{u}V_{u}}{\left(t_{c}\;-\;t_{p}\right)}.$$

In this formula, UER_avg_ is the average urinary excretion rate between the current void and the previous void, *C*_*u*_ is the measured metabolite concentration in the urine, *V*_*u*_ is the volume of the void, *t*_*c*_ is the time of the current void, and *t*_*p*_ is the time of the previous void [1]. The fourth variable held the body weight of the child in kilograms. The fifth variable denoted the route of the exposure and scenario, where 1 is an inhalation exposure, 2 is a dermal exposure, and 3 through 7 are bolus events. In this analysis, 3 was the first morning void on the second day, 4 was the lunchtime void, 5 was the before bedtime void on the first day, and 6 was the dinnertime void on the second day. The sixth variable held the start time of the exposure event on a 24-hour clock with 0 at midnight before the exposure event. The seventh variable held the end time of the exposure event. However, for the current analysis, this variable was not used since the exposures that were analyzed were bolus events. The eighth variable held the time of the previous urine void before the exposure event on a 24-hour clock. The ninth variable held the time of the current urine void sample before the exposure event on a 24-hour clock.

#### 2.2.2. Data Processing

Once input files were created, the SPK model was run using Matlab version 7.0.1. Each input into the simple SPK model was able to calculate an estimated absorbed dose for CPF by fitting the input variables to a generalized dose absorption curve. It then calculated the absorbed dose by finding the area under the curve over the course of a day. Due to the construction of the script, the model was only able to compute one dose estimate from one exposure rather than from a collection of exposures. Using the output from the SPK model script, we composited four spot urine samples in three different ways in order to estimate the daily absorbed doses of CPF in three different scenarios.

For the first composite (SPK I) scenario, only TCPY concentrations from the first morning void were used to calculate the dose estimate. This void was suggested to be an accurate measure of TCPY metabolite because it was assumed that TCPY concentrations in the urine were 0 after the before bedtime void [20]. Urine would then be formed and held during the night and voided in the morning, which would give the sample greater accuracy in determining metabolite concentrations since a long period of time had elapsed before the void.

For the second composite (SPK II) scenario, dose estimates from the before bedtime void and the first morning void were averaged normalized by the volume of each void. This was done because the SPK model was only able to analyze one sample at a time rather than taking a collection of samples and computing a dose from them. In this calculation, it was assumed that metabolite concentrations from the before bedtime void and the first morning void were both due to exposure from dinner on the first day. Since metabolite concentrations were assumed to be caused by the same exposure event (dinner event), they should compute to the same absorbed dose. Therefore, the two dose estimates were averaged to provide a fair measure based on both outputs.

In order to determine the times used to calculate the urinary excretion rate for the before bedtime sample of SPK II, we used the volume of the void sample to estimate the time that the urine was allowed to collect in the bladder. This estimate was based on the average daily void volume of children of the same age and gender over a 24-hour period [21]. By dividing the sample void volume by the average daily void volume, the time that the urine was allowed to collect in the bladder was estimated using (2), in which (*t*_*c*_ − *t*_*p*_) is the difference in hours from the time of the current void (*t*_*c*_) to the time of the previous void (*t*_*p*_), *V*_*u*_ is the volume of the urine sample, and V_avg24_ is the volume of the average daily void over the span of 24 hours:

(2) $$\left(t_{c}-t_{p}\right)\approx \left(\frac{V_{u}}{{\text{V}}_{\text{avg24}}}\right)*24$$

For the third composite (SPK III) scenario, dose estimates from the before bedtime void on the first day, and the first morning void, the lunchtime void, and the dinner void on the second day were averaged to reach a single absorbed dose estimate. For this calculation, the beginning exposure time for the lunch and dinner voids on the second day was set at dinner time on the first day. We assumed that part of the urinary metabolite measurements from the lunchtime and dinner voids on the second day may come from dinnertime exposure on the first day. Since absorbed dose estimates could not be calculated using more than one exposure, a composite meal exposure was created using urinary metabolite measurements from the before bedtime void on the first day, the first morning void, the lunchtime void, and the dinnertime void on the second day. The time of the composite meal exposure was set at dinnertime on the first day. The results from the four estimates were then averaged to get an overall absorbed dose estimate. Again, the times used to calculate the urinary excretion rate were estimated from dividing the sample void volume by the average daily void volume using (2).

## 3. Results and Discussion

The PBPK model was developed to quantitatively integrate the physiological, metabolic, and biochemical factors associated with ADME. Although the initial model validation has proven the accuracy of predicting CPF exposures, it was found later that the effectiveness of the PBPK model was highly dependent on the model parameters and the limitation of the input exposure data [2]. This limitation has been highlighted in a recent study in which the PBPK model failed to predict TCPY excretion as compared to TCPY levels measured in urine collected from study subjects [11]. Such failure exposes the vulnerability of employing PBPK models in risk assessment without sufficient knowledge or assurance of the quality of exposure data that are used as PBPK model inputs. Therefore, we were prompted to develop this simple pharmacokinetic model in order to improve the ability for estimating absorbed dose using urinary metabolite data as the model inputs. Should TCPY levels measured in urine be considered the gold standard in reflecting the exposure to CPF (by subtracting the preformed portion of TCPY), the use of urinary TCPY data in the pharmacokinetic analysis would provide more reliable dose estimates of CPF than using aggregate exposure data.

**Figure 2:**
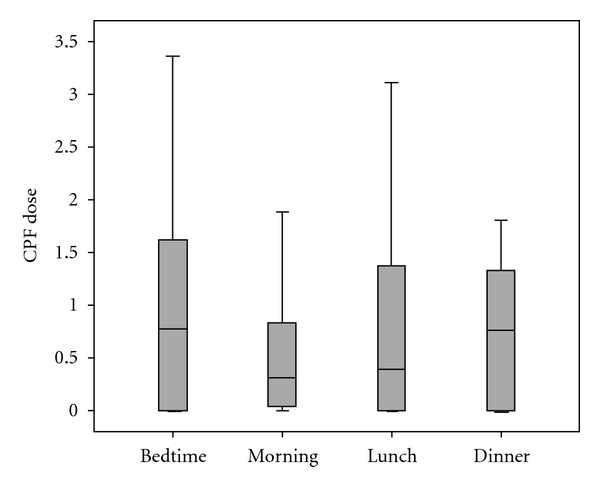
The dose estimates (CPF_DOSE, *μ*g/kg/day) for chlorpyrifos using 3,5,6-trichlorpyridinol concentrations in the spot urine samples collected at before bedtime in Day 1 and the first morning, lunch, and dinner voids in Day 2 as the SPK model inputs.

The SPK model simulation yielded to a total of 88 CPF dose estimates, separated by summer and fall seasons. The dose estimates resulted from the first morning void measurements appeared to be lower than but not significantly different to those using before bedtime, lunch, or dinner voids (Figure 2). Those estimates were modeled assuming that the exposure to CPF occurred during dinner meal (at 7 pm) on the first day. Knowing that the biological half-life for orally ingested CPF is approximately 16 hours [23], the estimated absorption curve for the first morning void may mostly reflect the intake of CPF from dinner last night. Dose estimates for spot urine samples collected later in the 2nd day were increasingly larger, suggesting additional CPF exposures from earlier in the day (breakfast and lunch). Dose estimates from before bedtime void samples were likely to be representative of CPF exposures that occurred throughout the first day rather than intakes from dinner, as was modeled.

The disparity among the dose estimates using spot urinary TCPY measurements in different time points raises the concern of the validity of using single spot urinary measurement as the basis of dose estimation in risk assessment. The magnitude of such uncertainty would be considerably larger under the circumstance in which spot urine samples are collected at different time points within a predetermined time period (e.g., 24 hours) from individuals. For instance, the National Health and Nutrition Examination Survey (NHANES) conducted by the Center for Disease Control and Prevention (CDC) collected single spot urine samples from subjects based on their appointments. If TCPY data from NHANES were used in the SPK model simulation, the interpretation and the conclusions of the estimated doses for CPF in NHANES subjects should therefore be cautiously made. Georgopoulos et al. [13] also raised this issue in their case study involving the use of a physiologically based toxicokinetic modeling in conjunction with numerical “inversion” techniques for reconstructing CPF exposure using TCPY data measured in the National Human Exposure Assessment Survey (NHEXAS). As authors stated “Although the NHEXAS data set provides a significant amount of supporting exposure-related information, especially when compared to national studies such as the NHANES, this information is still not adequate for detailed reconstruction of exposures under several conditions,” as demonstrated in the paper.

**Figure 3:**
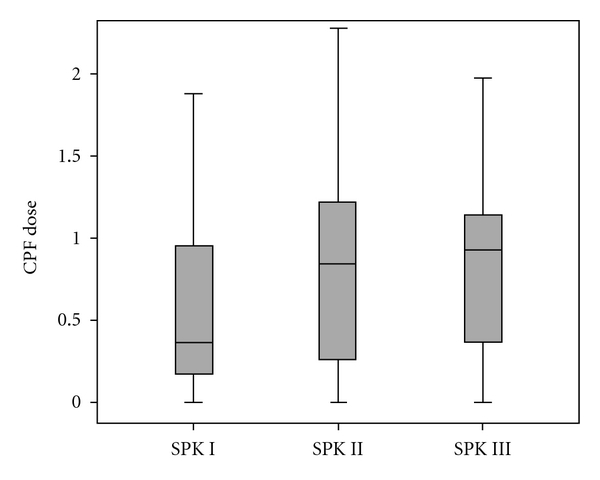
The dose estimates (CPF_DOSE, *μ*g/kg/day) for chlorpyrifos using urinary 3,5,6-trichlorpyridinol concentrations in three different spot urine sample composite methods; SPK_I, SPK_II, and SPK_III.

We estimated the daily absorbed CPF doses for individual children using three different composites of the 4 spot urine biomarker measurements (Table 1). Dose estimates for SPK I, II and III were not significantly different (one-way ANOVA); however, the box plot in Figure 3 showed that the median dose estimate from SPK I model simulation are lower than those of SPK II and III, consistent to the trend for the individual void simulations. While this may suggest that dose estimates between models reflect similar CPF exposure among subjects, it is also possible that the similarity among dose estimates is merely the result of the dose estimation being based on shared data. For instance, SPK II was based on the average between SPK I and the dose estimates using the before bedtime voids, and SPK III was based on the estimates that comprised the SPK II estimate and estimates from the lunch and dinner samples. The SPK II dose estimates seemed to be more accurately representative of the daily exposure to CPF simply because it takes into account the excretion of TCPY in before bedtime voids in day 1 and the first morning voids in day 2. The additional CPF exposure between lunch and dinner, if any, captured in the SPK III dose estimates did not significantly increase the overall daily dose estimates would support the validity of using SPK II as the benchmark for daily CPF absorbed dose. Comparing the dose estimates by seasonality and the community where children lived in 1998, we found that children living in urban/suburban have higher CPF exposure than those lived in agricultural community, and their CPF exposure was higher in fall than in summer season.

The SPK dose estimates for individual children participating in the 1998 study were significantly higher than those using the conventional PBPK model approaching, as published earlier [11]. The highest PBPK predicted CPY dose of 2.3 *μ*g/kg/day resulted from the consumption of a food item containing 350 ng/g of CPF remained as the highest in the SPK I, II, and III simulations with the estimated CPF doses of 9.1, 5.5, and 3.7 *μ*g/kg/day, respectively (Table 2). The striking difference of dose estimates between these two pharmacokinetic models may be primarily due to the differences of input data. The use of aggregate exposure assessment as the input variables in the PBPK models targeted at chemicals with short biological half-lives, such as CPF, is prone to significant spatial and temporal variations associated with exposures that would lead to inaccurate outcome measurements. This is evident by the fact that the majority of the data collected from the environment (Figure 1) where the children lived were nondetectable for CPF residue, while spot urine samples collected from those same children frequently contained TCPY. By taking into account the degradation of CPF in the environment (or in foods) in the PBPK model simulation as described in previous studies [11, 24, 25], the PBPK model predicted that TCPY excretion was still not within a reasonable range of accuracy to the measured TCPY levels which are used in the SPK model simulation. It is apparent that the 24-hr aggregate exposure assessment is not capable of capturing CPF exposure in those children. This leads to the serious doubt of the validity of applying aggregate exposure measurements to the PBPK model simulation and subsequently in the risk assessment. The use of urinary TCPY data in the SPK model simulation intuitively provided a plausible alternative to PBPK model in reconstructing the absorbed CPF dose. In theory, the dose estimates resulting from either PBPK or SPK approach should yield to numerical values that are not significantly different to each other, particularly in this case in which both models were constructed using identical parameters, and the input data were collected from a study that is designed specifically for validating the PBPK model simulation. However, the problem of significant under estimation of CPY doses using the traditional PBPK, as identified in this paper, would be even more prevalent and dramatic under other circumstances in which less-structured environmental exposure data are collected and used as the PBPK model inputs.

## 4. Study Limitations

Similar to other PBPK model applications, assumptions are needed in the SPK models in order to facilitate the model simulation. The unique assumptions for SPK model simulation include the prior knowledge of the predominant exposure pathway (such as dietary intake in this project) and the default time of exposure (dinner in Day 1). Without detail information of the exposure, those assumptions have to be made either arbitrarily (time of exposure) or by interpretation of study observations (dietary ingestion). A mischaracterization of the predominant exposure pathway (such as dietary intake versus inhalation) will lead to completely different SPK simulation and outcomes. 

Due to the limitation in constructing the script files, the SPK model is only able to compute one dose estimate from one urinary biomarker data input, instead of from a series of urinary biomarker data input within 24 hours. In order to overcome this limitation, we used the average dose estimates in SPK II and SPK III to incorporate more urinary biomarker data that is related to the interest of a specific exposure event in the simulation. It is likely that we would introduce unknown uncertainties to the overall simulation because urinary TCPY levels measured in the dinner time may include CPF exposure in Day 2 which is not the interest of the analysis. Although the magnitude of such uncertainty is relatively minimal in this study, by further examining the data for SPK II and SPK III, we acknowledge the existence of such uncertainty.

## Supplementary Material

The following information illustrates a step-by-step instruction of preparing the Matlab scrips, including the input data file specification and the model simulation commends, used in the simple pharmacokinetic model. Also included is the description of output data files from the Matlab model simulation runs.

## Figures and Tables

**Table 1:** Descriptive statistics for the estimated daily dose (*μ*g/kg/day) of chlorpyrifos in thirteen children ages 2–5 using the simple pharmacokinetic model.

Overall	SPK I	SPK II	SPK III
Mean (St. Dev.)	0.98 (1.95)	0.97 (1.19)	0.87 (0.81)
Median	0.37	0.82	0.92
*N*	21	23	26
95% confidence interval (lower, upper)	(0.09, 1.86)	(0.43, 1.51)	(0.51, 1.24)
Min–max	(0, 9.13)	(0, 5.5)	(0, 3.65)

Summer season			

Mean (St. Dev.)	0.55 (0.58)	0.8 (1.0)	0.73 (0.68)
Median	0.34	0.27	0.52
*N*	10	11	13
95% confidence interval (lower, upper)	(0.19, 0.91)	(0.24, 1.37)	(0.37, 1.09)
Min–max	(0, 1.89)	(0, 3.36)	(0, 2.5)

Fall season			

Mean (St. Dev.)	1.3 (2.53)	1.32 (1.42)	1.13 (0.9)
Median	0.42	0.97	0.94
*N*	11	12	13
95% confidence interval (lower, upper)	(0, 2.67)	(0.55, 2.09)	(0.66, 1.6)
Min–max	(0.05, 9.13)	(0.2, 5.5)	(0.23, 3.65)

Urban/suburban children			

Mean (St. Dev.)	0.48 (0.52)	0.67 (0.64)	0.63 (0.55)^1^
Median	0.35	0.48	0.45
*N*	11	12	12
95% confidence interval (lower, upper)	(0.19, 0.77)	(0.31, 1.03)	(0.32, 0.94)
Min–max	(0.04, 1.88)	(0.02, 2.27)	(0.01, 1.97)

Agricultural children			

Mean (St. Dev.)	1.53 (2.73)	1.51 (1.58)	1.19 (0.92)^1^
Median	0.88	1.06	1.04
*N*	10	11	14
95% confidence interval (lower, upper)	(0.1, 2.96)	(0.91, 2.11)	(0.68, 1.7)
Min–max	(0, 9.12)	(0, 5.5)	(0, 3.65)

^1^ Marginally significantly different (one-way ANOVA, *P* = 0.077).

**Table 2:** The dose estimates (*μ*g/kg/day) for chlorpyrifos using PBPK, SPK I, SPK II and SPK III model simulations.

Subject ID^1^	Season	Body wt. (kg)	PBPK	SPK I^2^	SPK II^3^	SPK III^4^
R1	Summer	15.5	0.004	0.546	0.273	1.140
R2	Summer	21.4	0.003	0.281	0.246	0.380
R3	Summer	14.5	0.004	0.043	0.022	0.014
R4	Summer	16.8	0.003	0.831	1.439	0.987
R5	Summer	13.4	0.004	0.338	0.213	0.158
R6	Summer	19.6	0.003	n.a.^ 5^	0.840	0.520
S1	Summer	17.3	0.003	0	0	0
S2	Summer	18.2	0	0.224	0.112	0.488
S3	Summer	15	0	n.a.^ 5^	3.357	2.497
S4	Summer	15.5	0	1.881	1.450	1.282
S5	Summer	16.8	0.004	n.a.^ 5^	n.a.^ 5^	0.326
S6	Summer	22.7	0.440	0.808	0.820	0.672
S7	Summer	14.5	0	n.a.^ 5^	n.a.^ 5^	1.056
R1	Fall	15.5	0.004	1.881	2.274	1.973
R2	Fall	21.4	0.003	0.051	0.204	0.229
R3	Fall	14.5	0.012	0.347	0.980	0.944
R4	Fall	16.8	0.003	0.366	0.541	0.603
R5	Fall	13.4	0.018	0.082	0.483	0.368
R6	Fall	19.6	0.005	0.476	0.476	0.250
S1	Fall	17.3	2.302	9.125	5.496	3.647
S2	Fall	18.2	0.003	0.172	0.949	1.110
S3	Fall	15	0.004	1.060	1.060	1.219
S4	Fall	15.5	0	0.956	1.260	1.017
S5	Fall	16.8	0	1.028	1.184	0.922
S6	Fall	22.7	0.001	0.041	0.969	0.934
S7	Fall	14.5	0.006	n.a.^ 5^	n.a.^ 5^	1.513

^1^“R” for children living in agricultural community and “S” for children living in urban/suburban community.

^2^Significantly different to PBPK dose estimates (paired *t*-test, *P* < 0.001).

^3^Significantly different to PBPK dose estimates (paired *t*-test,  *P* = 0.02).

^4^Significantly different to PBPK dose estimates (paired *t*-test, *P* < 0.001).

^5^Missing data due to missing spot urine samples.
