# Supplementary material for: Reconstructing Organophosphorus Pesticide Doses Using the Reversed Dosimetry Approach in a Simple Physiologically-Based Pharmacokinetic Model
Source: J Toxicol. 2012 Feb 1;2012:131854. doi: 10.1155/2012/131854 (PMC3306923; doi:10.1155/2012/131854)
Supplement: Supplementary file 1 — The following information illustrates a step-by-step instruction of preparing the Matlab scrips, including the input data file specification and the model simulation commends, used in the simple pharmacokinetic model. Also included is the description of output data files from the Matlab model simulation runs. [file 131854.f1.docx]

**Supplemental Information**

*Files Required by the Simple PK (SPK) Model Matlab Script*

There are 5 files that are required to run the SPK Model. These files must be included in the same directory and are described below.

solveTCP.M – The actual SPK Model that is run to calculate the absorbed doses.

solve_inhale.m – File that contains the function used to calculate the inhalation dose.

derm_rate.m – File that contains the function used to calculate the dermal dose.

solveoral.m – File that contains the function used to calculate the oral dose.

spkin.xls – Excel file that contains the input data for the model.

*Input Data File Specifications*

The input data for the SPK model is held in an Excel file containing data in 9 columns. The columns to the data file are described below.

Column 1 (houseid): Study ID and study period.

Column 2 (agdist): Whether or not the subject lived in an agricultural area.

0 – subject did not live in an agricultural area.

1 – subject lived in an agricultural area.

Column 3 (uer): Urinary excretion rate of metabolite in units of mmol/hr.

Column 4 (weight): Subject’s body weight in kilograms.

Column 5 (route): Route of exposure. The routes are enumerated as follows

1 – inhalation exposure

2 – dermal exposure

3 – first morning void

4 – lunchtime void

5 – dinnertime void

6 – ‘take home’ events modeled as a bolus exposure (same as routes 3-5)

7 – ‘take home’ events modeled as a bolus exposure (same as routes 3-5)

Column 6 (ctimebeg): Time for beginning of exposure or time of bolus event in hours from midnight on the day of the exposure.

Column 7 (ctimeend): Time for end of exposure in hours from midnight on the day of the exposure. Ignored in bolus exposures (routes 3-7)

Column 8 (tprev): Time for previous void in hours from midnight on the day of the exposure.

Column 9 (tvoid): Time for the current void in hours from midnight on the day of the exposure.

An example row of data is included below.

| **houseid** | **agdist** | **uer** | **weight** | **route** | **ctimebeg** | **ctimeend** | **tprev** | **tvoid** |
| --- | --- | --- | --- | --- | --- | --- | --- | --- |
| 6.1 | 0 | 5.9E-07 | 15.5 | 5 | 18 | 19 | 18 | 21 |

For this sample, labeled 6.1, the subject did not live in an agricultural area (agdist = 0). The urinary excretion rate for the metabolite in the sample was calculated at 5.9 * 10^-7^ mmol/hr. The subject weighed 15.5 kilograms and the sample was a dinnertime void (route = 5). The beginning time of the exposure was 6:00 PM (ctimebeg = 18), and the exposure ended at 7:00 PM (ctimeend = 19). The time of the void previous to the current sample void was at 6:00 PM (tprev = 18), and the time of the current void was at 9:00 PM (tvoid = 21).

*Running the SPK Model Matlab Script*

1. Open Matlab program (double click Matlab icon or run from start menu).
2. Open Current Directory window. This should be open on the left side of the screen. If this window is not open, then it can be selected by going to the Desktop menu item on the top of the program, and clicking Current Directory.
3. In the Current Directory window, navigate to the directory where the files for the SPK Model script.
4. Right-click on the SPK Model script file (solveTCP.M) and click Run.

*Output Data File Description*

The SPK Model script outputs the absorbed dose into an Excel file named spkout.xls. The columns for this file are described below.

Column 1 (houseid): Study ID and study period.

Column 2 (route): Route of exposure. The routes are enumerated as follows

1 – inhalation exposure

2 – dermal exposure

3 – first morning void

4 – lunchtime void

5 – dinnertime void

6 – ‘take home’ event modeled as a bolus exposure (same as routes 3-5)

7 – ‘take home’ event modeled as a bolus exposure (same as routes 3-5)

Column 3 (dose): Absorbed dose estimate for parent compound. For inhalation and

dermal routes, the units are in mmol/hr. For bolus events, the units are in mmol.

Column 4 (weight): Subject’s body weight in kilograms.
